# Angiopoietins Modulate Endothelial Adaptation, Glomerular and Podocyte Hypertrophy after Uninephrectomy

**DOI:** 10.1371/journal.pone.0082592

**Published:** 2013-12-18

**Authors:** Wen Chih Chiang, Chun Fu Lai, Chi Ting Su, Wei Hao Peng, Ching Fang Wu, Fan Chi Chang, Yi Ting Chen, Shuei Liong Lin, Yung Ming Chen, Kwan Dun Wu, Kuo Shyan Lu, Tun Jun Tsai

**Affiliations:** 1 Department of Internal Medicine, National Taiwan University Hospital, Taipei, Taiwan; 2 Department of Internal Medicine, National Taiwan University Hospital Yunlin Branch, Yunlin County, Taiwan; 3 Graduate Institute of Anatomy and Cell Biology, College of Medicine, National Taiwan University, Taipei, Taiwan; 4 Department of Internal Medicine, National Taiwan University Hospital Zhudong Branch, Hsinchu County, Taiwan; 5 Department of Internal Medicine, E-Da Hospital, Kaohsiung City, Taiwan; 6 Graduate Institute of Physiology, College of Medicine, National Taiwan University, Taipei, Taiwan; Fondazione IRCCS Ospedale Maggiore Policlinico & Fondazione D’Amico per la Ricerca sulle Malattie Renali, Italy

## Abstract

Glomerular capillary remodeling is an essential process in the development of glomerular hypertrophy. Angiopoietins, which are important regulators in angiogenesis, plays a role in the development of glomerulus during embryogenesis. Here, we evaluated the influence of angiopoietin on glomerular components and hypertrophy after uninephrectomy in adult male BALB/c mice. The actions of angiopoietin 1 or 2 were systemically antagonized by the subcutaneous administration of antagonists. We observed that the angiopoietin system was activated after uninephrectomy, and that the blockade of angiopoietin 1 or 2 decreased the activation of the angiopoietin receptor—tyrosine kinase with Ig and EGF homology domains-2—and attenuated the development of glomerular and podocyte hypertrophy. The increase in endothelial density staining (anti-CD31) following uninephrectomy was also reversed by angiopoietin 1 or 2 blockades. Glomerular basement thickness and foot process width were observed to decrease in the angiopoietin blockade groups. These changes were associated with the down regulation of the expression of genes for the glomerular matrix and basement membrane, including collagen type IV α1, collagen type IV α2, collagen type IV α5, and laminin α5. Thus, angiopoietin 1 or 2 may play an important role in the development of glomerular hypertrophy after uninephrectomy. A blockade of the angiopoietin system not only influenced the endothelium but also the podocyte, leading to diminished gene expression and morphological changes after uninephrectomy.

## Introduction

Glomerular hypertrophy is a compensatory mechanism adopted by residual glomeruli in response to the loss of functional nephrons in chronic kidney disease; it is also a pathological consequence of glomerular diseases, such as diabetes. It is believed that glomerular hypertrophy is associated with the development of glomerulosclerosis during the pathological processes involved in chronic kidney disease. The development of glomerular hypertrophy includes an increase in the glomerular matrix along with hypertrophy and proliferation of component cells. Glomerular capillaries may detect changes related to renal parenchymal loss by sensing the increase in renal blood flow to accommodate the loss of functional nephrons, eventually leading to glomerular hypertrophy. The growth of glomerular capillaries after nephrectomy occurs by branching that makes new glomerular capillaries, instead of simply lengthening the existing capillaries. [1] In experimental diabetes and toxic nephropathy due to lithium as well, growth is accomplished by new capillary branching. [2], [3] Lengthening and branching of the capillaries are processes involved in both angiogenesis and blood vessel maturation. Regulation of angiogenesis and vascular maturation involve several signaling cascades that are driven by endothelial cell-specific growth factors and their receptors. These endothelial growth factors may also participate in the process of glomerular capillary remodeling in glomerular hypertrophy after the loss of functional nephrons. This concept has been previously demonstrated in several vascular endothelial growth factor (VEGF)-related studies as follows. The administration of anti-VEGF antibody in uninephrectomized mice was demonstrated to prevent glomerular enlargement and partially blocked renal growth. [4] Further, neutralizing VEGF also prevented glomerular hypertrophy in obese diabetic rats, [5] and in high protein-induced glomerular hypertrophy, the administration of anti-VEGF antibody similarly prevented the development of hypertrophy. [6]

In addition to VEGF, angiopoietins (Angpt 1 and 2) and their receptor, i.e., tyrosine kinase with Ig and EGF homology domains-2 (Tie2), are also involved in the process of vascular generation and maturation. Angpt 1 is produced by vascular mural cells, pericytes, and certain other cells, whereas Angpt 2 and Tie2 are expressed primarily by endothelial cells. [7], [8], [9], [10] In glomeruli, Angpt 1 is produced by podocytes. [11], [12], [13] Angpt 1 causes Tie2 auto phosphorylation, promoting vessel maturation via increased mural cell [14] and matrix [15] contacts along with reduced permeability. [16] Angpt 2 is a competitive antagonist that participates in the remodeling of immature blood vessels. [9] Several studies have revealed that the angiopoietin system may play a role in glomerular development in the embryonic and postnatal stages, for example, Yuan *et al.* observed increased Angpt 1 expression in the glomerulus during the embryonic and postnatal stages. The angiopoietin-Tie2 system is known to be activated during glomerular maturation. [17] Knocking out Angpt 1 expression in the embryonic stage disrupts glomerular maturation, resulting in glomerular capillary dilatation. [18] In the Thy1.1 glomerulonephritis model, Angpt 1 and Angpt 2 gene expression were markedly upregulated at day 6 of the diseased state when capillary restoration was noted to begin. [11] Thus, previous studies have indicated that angiopoietins may play a role in glomerular capillary remodeling in the normal as well as the diseased kidney.

Since capillary elongation and branching, which are observed in glomerular hypertrophy, are also processes involved in angiogenesis or capillary remodeling, angiopoietins may thus also play a role in the development of glomerular hypertrophy. To test this hypothesis, we antagonized the actions of Angpt 1 or Angpt 2 through the systemic administration of antagonists in uninephrectomized mice. A peptidobody “mL4-3”, an inhibitor of Angpt 1[19], [20], inhibits Tie2 phosphorylation *in vivo.* [21] Another peptidobody “L1-10” inhibits the partial agonist effect of Angpt 2 induced HUVEC Tie2 phosphorylation *in vitro* and reverse the antagonist effect of inhibition of Angiopoietin 2 on Tie2 phosphorylation in mouse adductor muscle *in vivo*. [22] These antagonists were used in this studies. The activation of the angiopoietin system and the changes in glomerular morphology, renal functions, and gene expressions occurring in response to this angiopoietin blockade were then evaluated in order to clarify the mechanisms by which angiopoietins modulate glomerular hypertrophy.

## Methods

### Animal model

Adult (5∼6-week-old) male BALB/c mice were anesthetized prior to surgery. Uninephrectomy was performed through a left lateral incision of the abdominal wall, and the left kidney was removed after renal vessel ligation. Sham mice underwent an abdominal wall incision without removal of the left kidney. For studying the expression of angiopoietins and Tie2, mice (N  =  6 for sham and nephrectomy groups each at each time point) were sacrificed at 2 weeks and 1, 2, and 3 months after nephrectomy. For evaluating the effects of the angiopoietin blockade, in addition to the sham group, the uninephrectomized mice were divided into three groups (N  =  12). Mice were injected subcutaneously with human IgG1 Fc (4 mg/kg), Angpt 1 antagonist (mL4-3, 4 mg/kg), or Angpt 2 antagonist (L1-10, 4 mg/kg) from 28 days after surgery and then every alternate day, until sacrifice on day 48 or 72. The mice kidneys were collected for pathological examination and RNA and protein preparation. Animals were housed in the animal center of the College of Medicine, National Taiwan University. The animal study protocol was approved by the Institutional Animal Care and Use Committee (IACUC) of the College of Medicine and College of Public Health, National Taiwan University.

### Cell culture

Immortalized mouse podocytes were maintained in permissive conditions at 33°C with γ-interferon supplement. [23] For experimental purposes, mouse podocytes were shifted to 37°C and cultured for 7∼14 days before harvest. Harvested cultured podocytes were subjected to RNA extraction, and mRNA expression was examined.

### Renal pathology and measurement of glomerular volume

The mice kidneys were fixed in formaldehyde and imbedded in paraffin, sectioned into 4-µm slices, and stained with periodic acid-Schiff and hematoxylin stains. To determine the average glomerular tuft volume (V_G_) for each kidney, pictures of at least 20 intact glomeruli with a vascular pole were taken under microscopy with 400× magnification. The mean glomerular random cross-sectional area (A_G_) was measured using FoveaPro4 software (Reindeer Graphics Inc., Asheville, NC, USA). V_G_ was then calculated as V_G_  =  (b/k)×(A_G_)^3/2^, where b  =  1.38 was the shape coefficient for spheres and k  =  1.1 was the size distribution coefficient. [24]

### Real-time quantitative PCR

The glomeruli of individual mice were isolated by gradually sieving with stainless steel sieves (250, 150, 75, and 38 µm), which resulted in 70∼80% pure glomeruli as judged by light microscopy inspection. RNA was extracted, subjected to DNase digestion (Qiagen, Valencia, CA, USA), and then reverse-transcripted using oligo-dT primers. Expression of genes was determined by real-time quantitative PCR using the ABI 7900 system (Applied Biosystems, Foster City, CA, USA). TaqMan gene expression assay kits for Angpt 1, and Angpt 2 and endothelial nitric oxide synthase (eNOS) were purchased from Applied Biosystems. The gene expression of laminin α5 and collagen type IV α1, α2, and α5 were analyzed using the SYBR green method. The primers used are listed in Table 1. The gene expression from each mouse was analyzed using the relative quantitative method. β-actin or the GAPDH gene was used as the internal control.

**Table 1:** Primers used in CYBR green real time quantitative PCR.

Laminin α5 forward	5′-ACC CAA GGA CCC ACC TGT AG-3′
Laminin α5 reverse	5′-TCA TGT GTG CGT AGC CTC TC-3′
Collagen type IV α1 forward	5′-AAA TTT CCA GGG ACC CAA AG-3′
Collagen type IV α1 reverse	5′-ACC TTT CAC ACC ACC AGG AG-3′
Collagen type IV α2 forward	5′-CCC TCC AGG TTT CCC TAC TC-3′
Collagen type IV α2 reverse	5′-TCC AGG TTG ACA CTC CAC AA-3′
Collagen type IV α5 forward	5′-CTC CAA CTT GTG GCA AAC CT-3′
Collagen type IV α5 reverse	5′-CTA GTG CCC ACT TGC TGA CA-3′

### Western blotting

The glomeruli were lysed with a RIPA buffer containing proteinase and phosphatase inhibitors. Certain amount of protein were subjected to SDS-PAGE electrophoresis, transferred to PVDF membrane, then blotted with anti-β actin, anti-phospho-Tie 2(Tyr992), or anti-Tie2 antibody. The degree of Tie2 receptor phosphorylation was evaluated with the anti-phopho-Tie2 to anti-Tie2 intensity ratio in Western blotting. The β actin was used as loading internal control.

### Immunofluorescence and immunohistochemical study of kidney tissue

Unfixed kidney or tissue perfused with 4% paraformaldehyde/PBS was sectioned into 4-µm thick slices. Fixed tissue sections were subjected to antigen retrieval with microwave treatment in a sodium citrate buffer. Then, tissue sections were permeabilized with 0.3% triton-X 100 in PBS, blocked with 5% normal goat or donkey serum, and probed with primary antibody (anti-synaptopodin, CD31, WT1, von Willebrand factor, and Tie2). After washing, tissue sections were probed with fluorophore-conjugated secondary antibodies; they were then mounted and subjected to fluorescence microscopy. In the immunohistochemical study, a peroxidase-conjugated linker was added after washing off the primary antibody. Staining was performed using DAB as a substrate, and the nucleus was counterstained with hematoxylin. The CD31 density index was calculated by dividing the CD31-positive staining area with the total glomerular area.

### Evaluation of podocyte hypertrophy, foot process width, and GBM thickness

Renal tissues were stained with anti-WT1 and synaptopodin antibodies, and secondary antibodies were bound with different fluorophores. Tissue sections were then observed under fluorescence microscopy. At least 15 glomeruli, wherein the vascular pole could be identified, were randomly selected from a kidney section for each mouse. WT1-positive cells located in the synaptopodin-positive area or proximal to the urinary space (WT1-positive/synaptopodin-positive cells) were defined as podocytes. The number of these cells in each glomerulus was counted, and the synaptopodin-positive area of each glomerulus was calculated by automated computer analysis using the FoveaPro4 program. The ratio of the synaptopodin-positive area to the number of WT1-positive cells was calculated as an index of podocyte hypertrophy. The number of podocytes was counted using the WT1-positive staining cells per glomerular surface area in each glomerulus. For electron microscopy, kidneys were fixed in a 4% glutaraldehyde/cacodylate buffer, and then processed according to standard procedure. Electron micrographs (×50000) were obtained for 5 glomeruli from each of 3 different mice We measured the following: (*1*) GBM thickness at 5 points on each photomicrograph along a selected length of GBM located over the most distal site from the mesangial pole, and (*2*) the foot process width for 5 consecutive typical foot (minor) processes.

### Materials and statistical analysis

Human IgG1 Fc fragments were purchased from Bethyl Laboratory Inc. (Montgomery, TX, USA ). L1-10 and mL4-3 (peptibodies) were provided by Amgen Inc. (Thousand Oaks, CA, USA). Tie2 and phospho-Tie2(Tyr992) antibodies were obtained from Merk Millipore (Billerica, MA, USA ). Synaptopodin antibody was purchased from Santa Cruz Biotechnology Inc. (Santa Cruz, CA, USA). Anti-CD31 antibody was obtained from eBioscience Inc. (San Diego, CA, USA). The WT1 antibody was purchased from Abnova (Taipei, Taiwan).

Data were expressed as mean ± S.E. Statistical analyses were carried out using GraphPad Prism (GraphPad Software, San Diego, CA, USA). The statistical significance was evaluated by one-way ANOVA with *P* less than 0.05 considered statistically significant..

## Results

### Angpt 1 and Angpt 2 antagonist administration attenuated glomerular and renal angiopoietin activation following uninephrectomy

**Figure 1:**
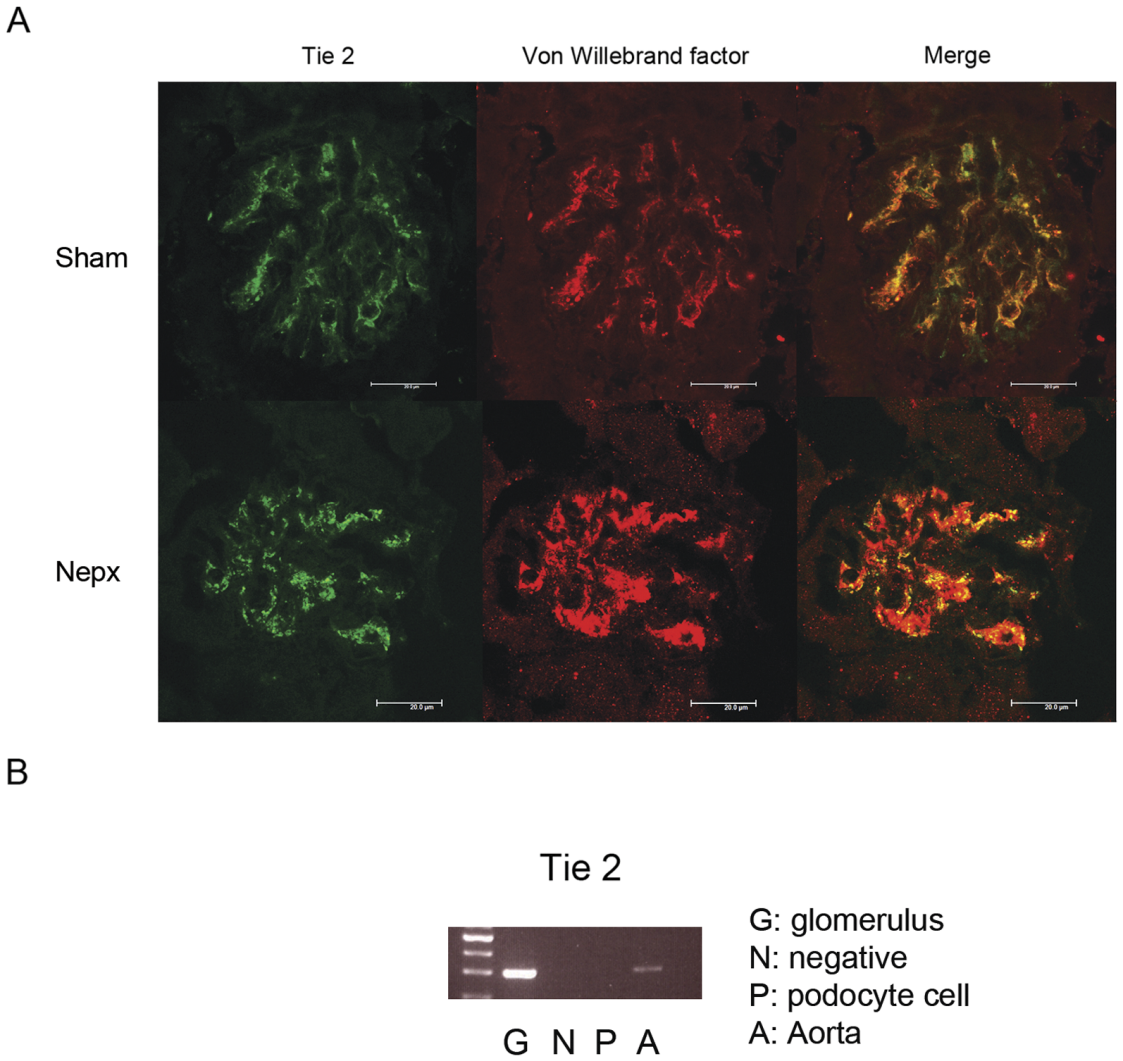
Expression of Tie2 receptor in glomerulus and podocyte. (A) Immunofluorescence study of the Tie2 receptor in mouse glomerulus. The staining of Tie2 (green) co-localized with endothelium (red, labeled as Von Willebrand factor) along the glomerular capillary in sham and uninephrectomized (Nepx) mice 2 months after the operation. (B) Expression of the Tie2 receptor in the 8 week-old normal mice glomerulus and cultured podocytes analyzed by the RT-PCR method. The glomerulus showed Tie2 mRNA expression, while the cultured podocyte showed no such expression. Messenger RNA from the normal mice aorta was used as a positive control. No cDNA was added to the PCR reaction in the negative control sample.

**Figure 2:**
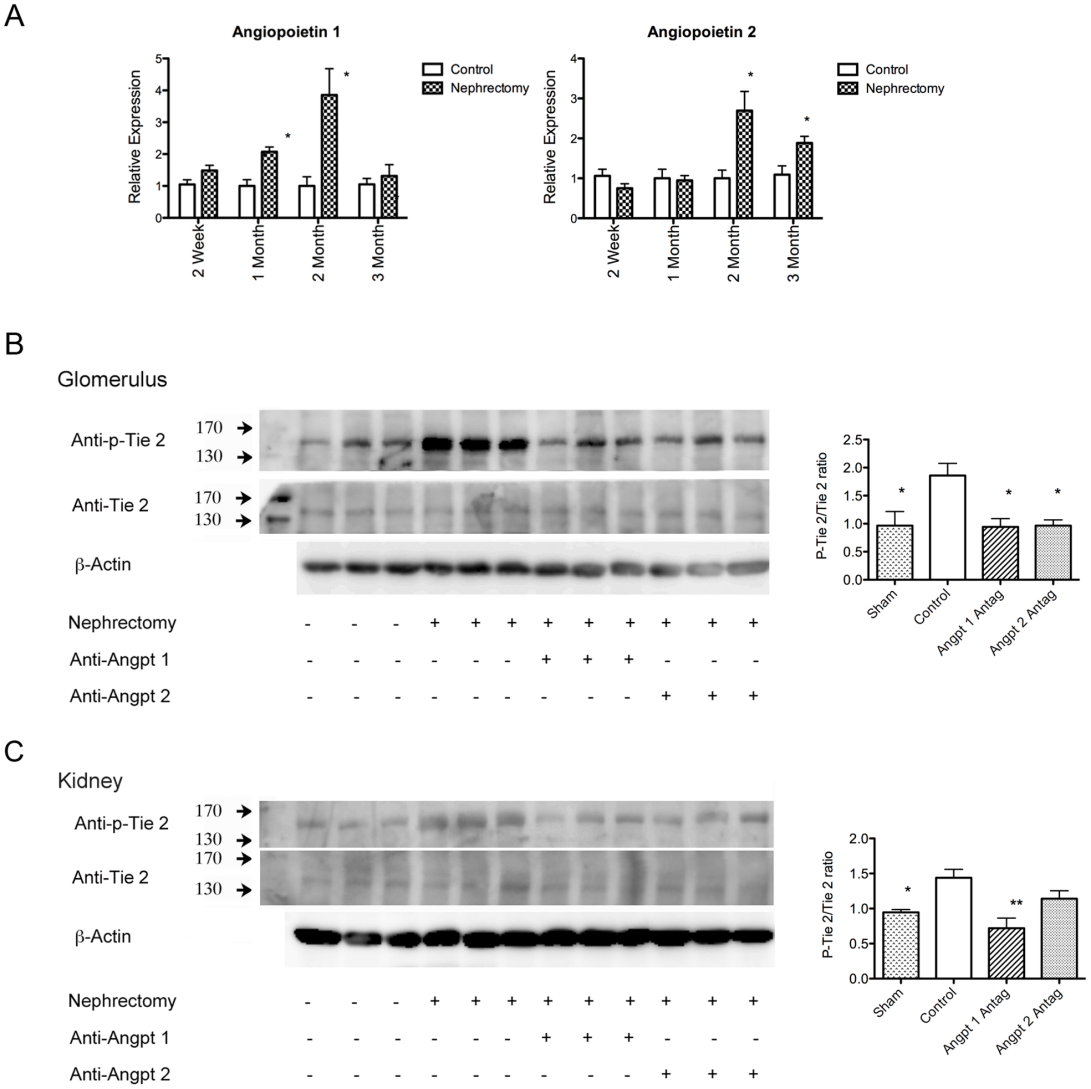
Activity of angiopoietin system after uninephrectomy and in each study group. (A) Glomerular Angpt 1 and Angpt 2 gene expression analyzed by real-time quantitative PCR. Angpt 1 began to increase at 1 month after uninephrectomy and reached the maximum level at 2 months after the operation. Angpt 2 was upregulated at 2 months after the operation. At 3 months after the operation, Angpt 2 levels began to decline but were still higher than those in the sham mice. (B) Glomerular Tie2 phosphorylation in different groups 2 months after uninephrectomy. The activation of the Tie2 receptor analyzed by Western blotting with anti-phospho-Tie2 antibody. Increased phosphorylation of the glomerular Tie2 receptor after uninephrectomy was attenuated by Angpt 1 or Angpt 2 blockade. Right panel showed the quantification of the ratio of phosphorylated Tie2 receptor to total Tie2 receptor (N = 3). This calculation indicated the proportion of phosphorylation in Tie2 receptor. (C) Whole kidney Tie2 receptor activation was also noted after 2 months of uninephrectomy. This activation was also attenuated by Angpt 1. (* *P*<0.05, **P<0.01)

In normal mice and uninephrectomized mice, the Tie2 receptor was mainly localized in the endothelium of the glomerulus. No Tie2 staining was observed in the other cells of the glomerulus. The cultured podocytes also did not express the Tie2 receptor (Figure 1). Upregulation of glomerular Angpt 1 gene expression was first observed at 1 month after uninephrectomy; it reached the maximum level at 2 months after the operation, and then decreased to the level observed in the sham mice at 3 months after the operation. The glomerular gene expression of Angpt 2 also was upregulated at 2 months after the operation; it then declined at 3 months after the nephrectomy (Figure 2A). The activation of the glomerular Tie2 receptor was evaluated by measuring the Tie2(Tyr992) phosphorylation of the receptor. Western blotting revealed that uninephrectomy activated the glomerular Tie2 receptor, while antagonizing Angpt 1 or Angpt 2 attenuated glomerular Tie2 receptor phosphorylation (Figure 2B). Administration of Angpt 1 antagonist also attenuated, while the Angpt 2 antagonist tended to attenuate, whole kidney Tie2 receptor activation induced by uninephrectomy (Figure 2C).

### The angiopoietin blockade prevented uninephrectomy-induced renal and glomerular hypertrophy

**Figure 3:**
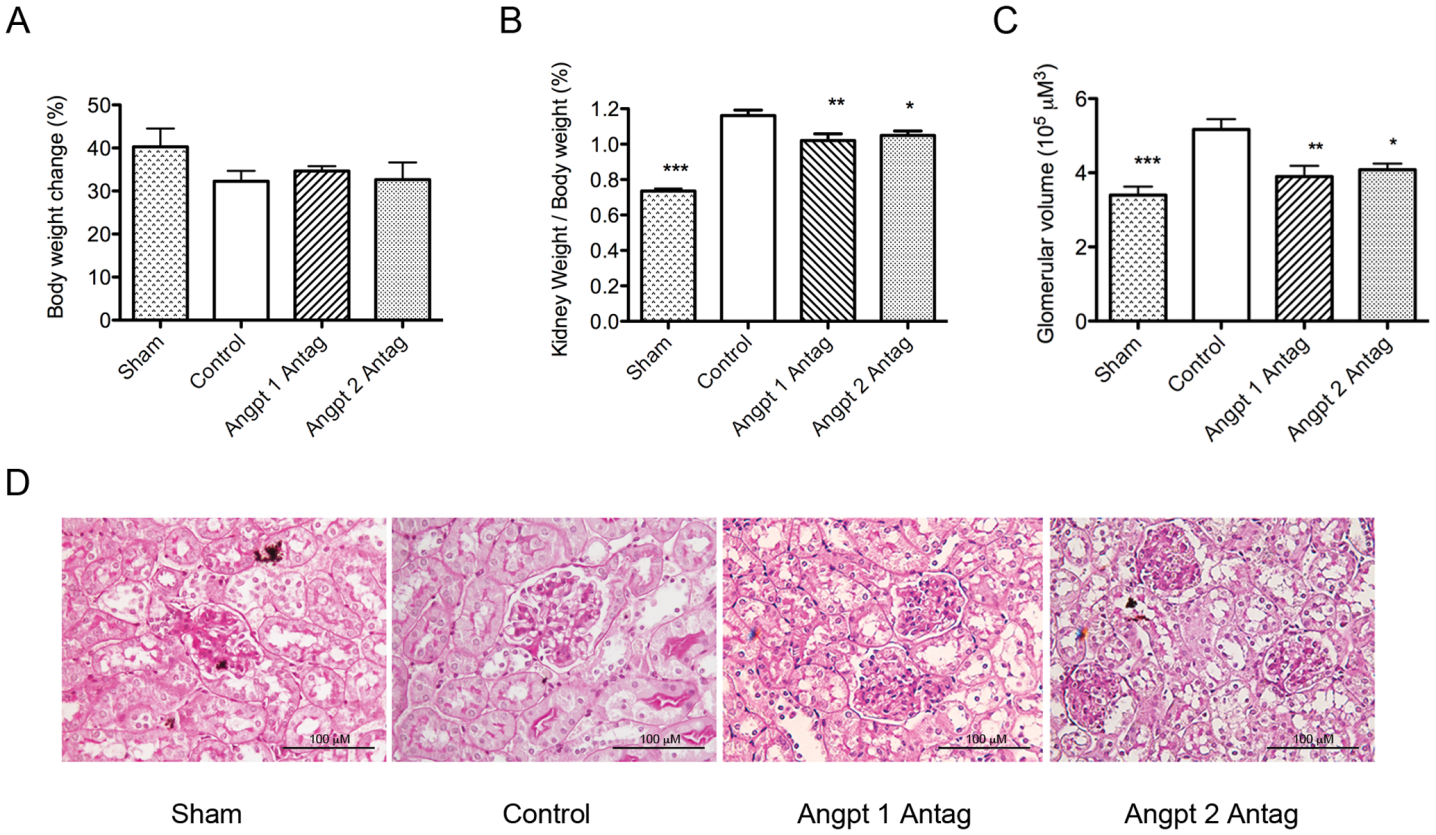
Body weight, kidney weight, and glomerular volume were evaluated at 3 months after uninephrectomy. (A) Change in body weight in each group. No significant difference was observed between the control, Angpt 1 and Angpt 2 antagonist groups. (B) Kidney/body weight percentage in each group. Uninephrectomy induced a significant increase in the kidney weight of the control group. Administration of Angpt 1 or Angpt 2 antagonist attenuated the increase in kidney weight. (C & D) Average glomerular volume and periodic acid-Schiff/hematoxylin staining in each group. Uninephrectomy induced a significant increase in the glomerular volume in the control group, while the administration of Angpt 1 or Angpt 2 antagonists attenuated this increase (* *P*<0.05, **P<0.01, ***P<0.001).

The Angpt 1 or Angpt 2 blockade did not significantly affect the body weight of the experimental mice; however, it attenuated the increase in kidney weight induced by uninephrectomy (Figure 3A and 3B, Table 2). Uninephrectomy induced a significant increase in glomerular volume, indicating the presence of glomerular hypertrophy. This hypertrophy was also attenuated by the Angpt 1 or Angpt 2 blockade (Figure 3C and 3D, Table 2). These findings indicated that the effects of the Angpt 1 or Angpt 2 blockade on renal or glomerular hypertrophy did not occur through inhibition of whole body growth but through the specific inhibition of renal growth. The administration of the Angpt 1 or Angpt 2 antagonist did not result in any proteinuria or renal function changes in the uninephrectomized mice (Table 2).

**Table 2:** Body weight change, kidney weight, degree of proteinuria, serum creatinine level and estimated glomerular volume in each group 3 months after uninephrectomy. (mean ± standard error).

	Body weight change (%)	Kidney weight/body weight (%)	Proteinuria [albumin/Cr (µg/mg)]	Serum creatinine (mg/dL)	Glomerular volume (µm^3^)
Sham	40.26±4.26	0.74±0.01***	15.01±6.80	0.23±0.03	3.400±0.227***
Control	32.38±2.42	1.16±0.03	18.71±3.80	0.26±0.04	5.168±0.280
Angpt 1 antagonist	34.67±1.09	1.02±0.04**	15.58±6.30	0.24±0.03	3.899±0.289**
Angpt 2 antagonist	32.64±3.99	1.05±0.03*	14.71±1.13	0.23±0.02	4.081±0.168*

*P*<0.05 compared to control.

** *P*<0.01 compared to control.

*** *P*<0.001 compared to control.

### The angiopoietin blockade attenuated the uninephrectomy-induced compensatory response of podocyte hypertrophy

**Figure 4:**
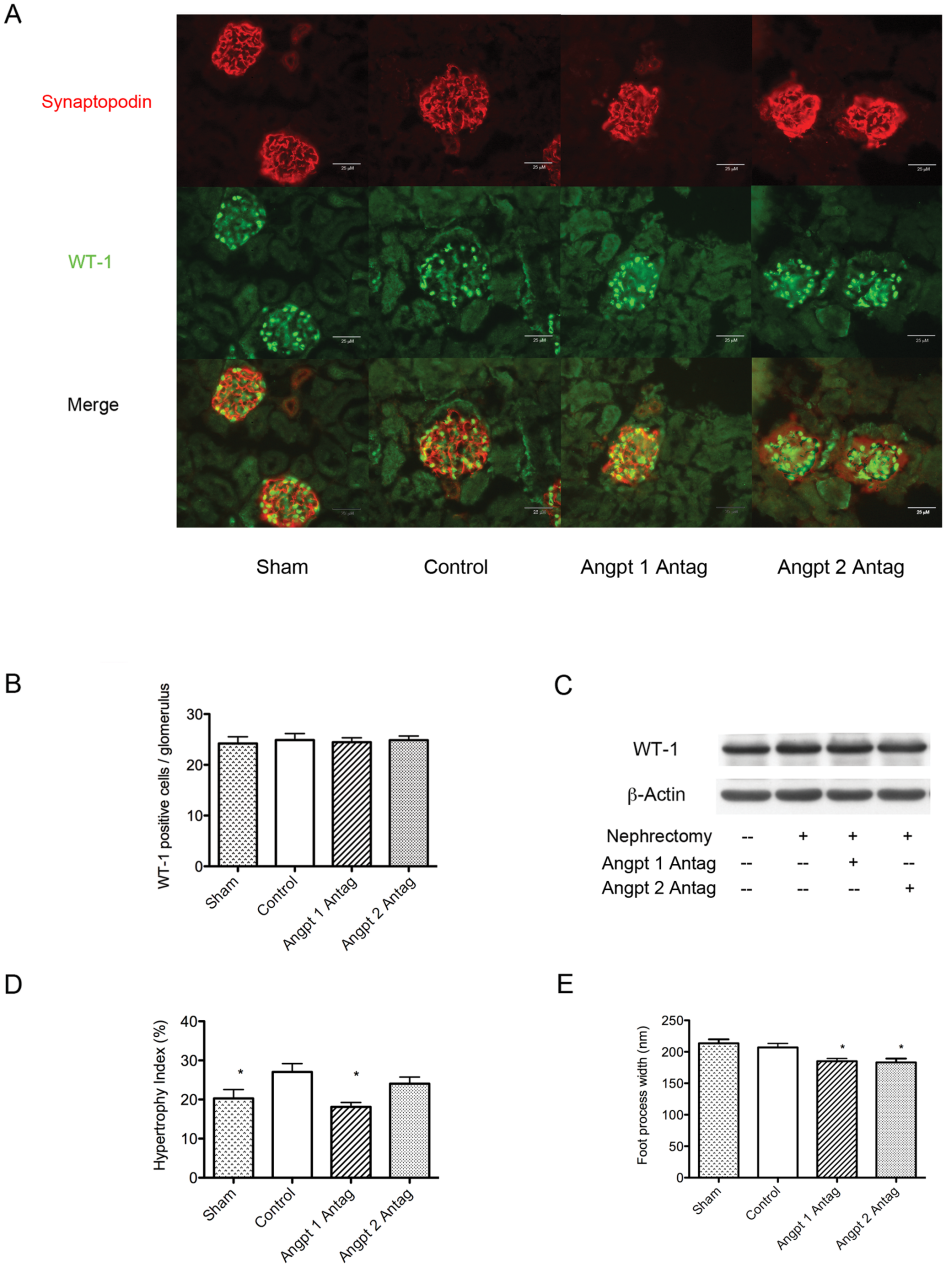
Podocyte evaluation at 3 months after uninephrectomy. (A) Immunofluorescence study of glomerular synaptopodin and WT1 in each group. (B) Average glomerular podocyte number (evaluated using the number of WT1-positive staining nuclei) in each group. No differences were noted among the groups. (C) Western blotting for glomerular WT1 protein expression in each group. No differences in WT1 expression were observed among the groups. (D) Podocyte hypertrophy index in each group. Uninephrectomy induced significant podocyte hypertrophy, while the administration of the Angpt 1 antagonist attenuated the degree of hypertrophy, while blocking Angpt 2 action only tended to attenuate hypertrophy. (E) Minor foot process width measured by electron microscopy. Uninephrectomy did not influence podocyte foot process width. The administration of Angpt 1 or Angpt 2 antagonists significantly decreased the podocyte foot process width in uninephrectomized mice (* *P*<0.05).

The development of glomerular hypertrophy is associated with lengthening and branching of the glomerular capillary loop and subsequent increased filtration area. [1], [2], [3] In order to cover the increased glomerular filtration surface, glomerular hypertrophy is typically accompanied by proliferation or hypertrophy of component cells. The podocyte number was therefore evaluated by counting the WT1-positive cells in the glomeruli; we observed that the podocyte number did not increase following uninephrectomy. Neither the antagonist against Angpt 1 nor Angpt 2 affected the number of podocytes (Figure 4A and 4B). WT1 protein expression was also not affected by uninephrectomy or by the administration of angiopoietin antagonists (Figure 4C). This finding indicated that podocyte number did not change after uninephrectomy or angiopoietin blockade. We then analyzed whether there was increased size of podocytes. The size of podocyte was evaluated with hypertrophy index. Uninephrectomy caused significant podocyte hypertrophy in the remaining kidney and this effect was reversed by the administration of antagonist of Angpt 1. Angpt 2 blockade tended to (but not statistically significant) attenuate podocyte hypertrophy induced by uninephrectomy (Figure 4A, 4D).

To clarify the manner in which podocyte hypertrophy covered the increased capillary surface area following uninephrectomy, the podocyte foot process width was evaluated by electron microscopy. The results revealed that foot process width was not increased after uninephrectomy, indicating that the podocyte protruded new or elongate its existing foot process instead of widening the previously existing minor foot process to compensate for the increased filtration area resulting from glomerular hypertrophy. Antagonizing the actions of Angpt 1 or Angpt 2 not only attenuated podocyte hypertrophy but further decreased the foot process width when compared with sham mice (Figure 4E, 6A), indicating that blocking the actions of Angpt 1 or Angpt 2 in uninephrectomized mice might result in a smaller foot process formation in podocytes. To cover a filtration surface similar to that in the sham mice, podocytes may need to elongate or protrude a greater number of foot processes in mice wherein Angpt 1 or Angpt 2 antagonists have been administered.

### The angiopoietin blockade inhibited the uninephrectomy-induced increase in endothelial marker CD31 density in the glomeruli

**Figure 5:**
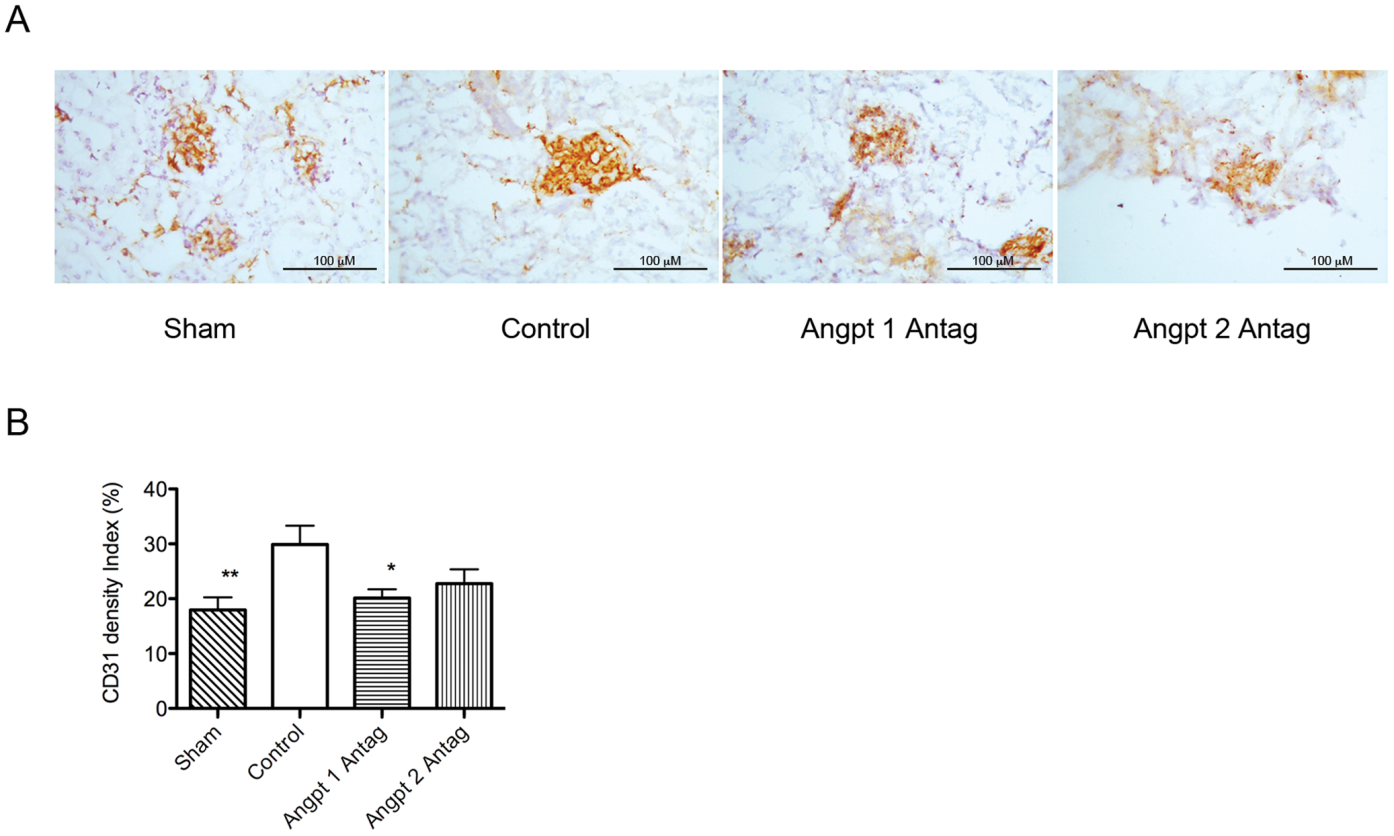
Glomerular endothelial density evaluation in each group. (A) Immunohistochemical study of the glomerular endothelial marker CD31 in each group 3 months after uninephrectomy. (B) Semi-quantitative analysis of the glomerular CD31 density in each group. The glomerular CD31 density was observed to be higher in the uninephrectomized than in the sham group. Blocking the action of Angpt 1 attenuated the increase in CD31 density induced by uninephrectomy. Blocking the action of Angpt 2 tended to attenuate the increase. (* *P*<0.05, **P<0.01).

Uninephrectomy-induced glomerular hypertrophy was accompanied by increased glomerular CD31 staining. This increase was also attenuated by the Angpt 1 blockade. Further, the Angpt 2 blockade only tended to (but not significantly) attenuate the increase in CD31 staining (Figure 5A, 5B).

**Figure 6:**
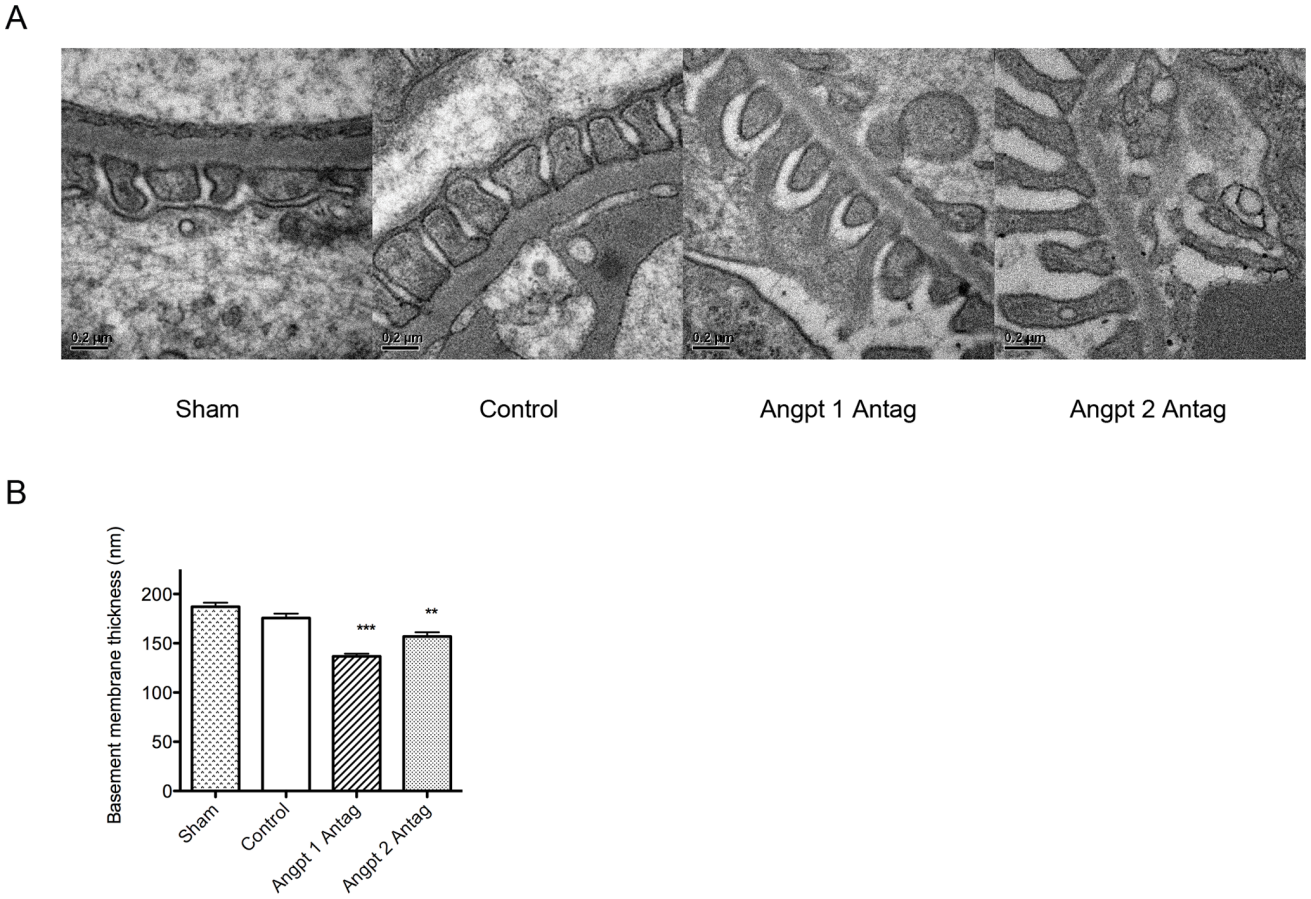
Filtration barrier structure examined with electron microscopy. (A) Foot processes and basement membrane in each group at 3 months after uninephrectomy. (B) Semi-quantitative analysis of glomerular basement membrane thickness. No difference was noted between the sham and uninephrectomized mice. Blocking the action of Angpt 1 or Angpt 2 significantly decreased the basement membrane thickness (**P<0.01, ***P<0.001).

The glomerular gene expression of eNOS was not influenced by uninephrectomy or the Angpt1 or Angpt 2 blockades (data not shown). Further, electron microscopy also did not reveal glomerular endothelial cell detachment or subendothelial changes in any of the groups in this study (data not shown).

### The angiopoietin blockade decreased average glomerular basement membrane thickness and basement membrane gene synthesis

Glomerular basement membrane is maintained by the endothelium and podocytes. As shown above, the angiopoietin blockade influenced the endothelium and podocytes. We therefore further evaluated the impact of the angiopoietin blockade on the glomerular basement membrane. Our results revealed that uninephrectomy did not cause any significant changes in the average basement membrane thickness. However, the Angpt 1 or Angpt 2 blockade caused a significant decrease in the basement membrane thickness in uninephrectomized mice (Figure 6A, 6B).

**Figure 7:**
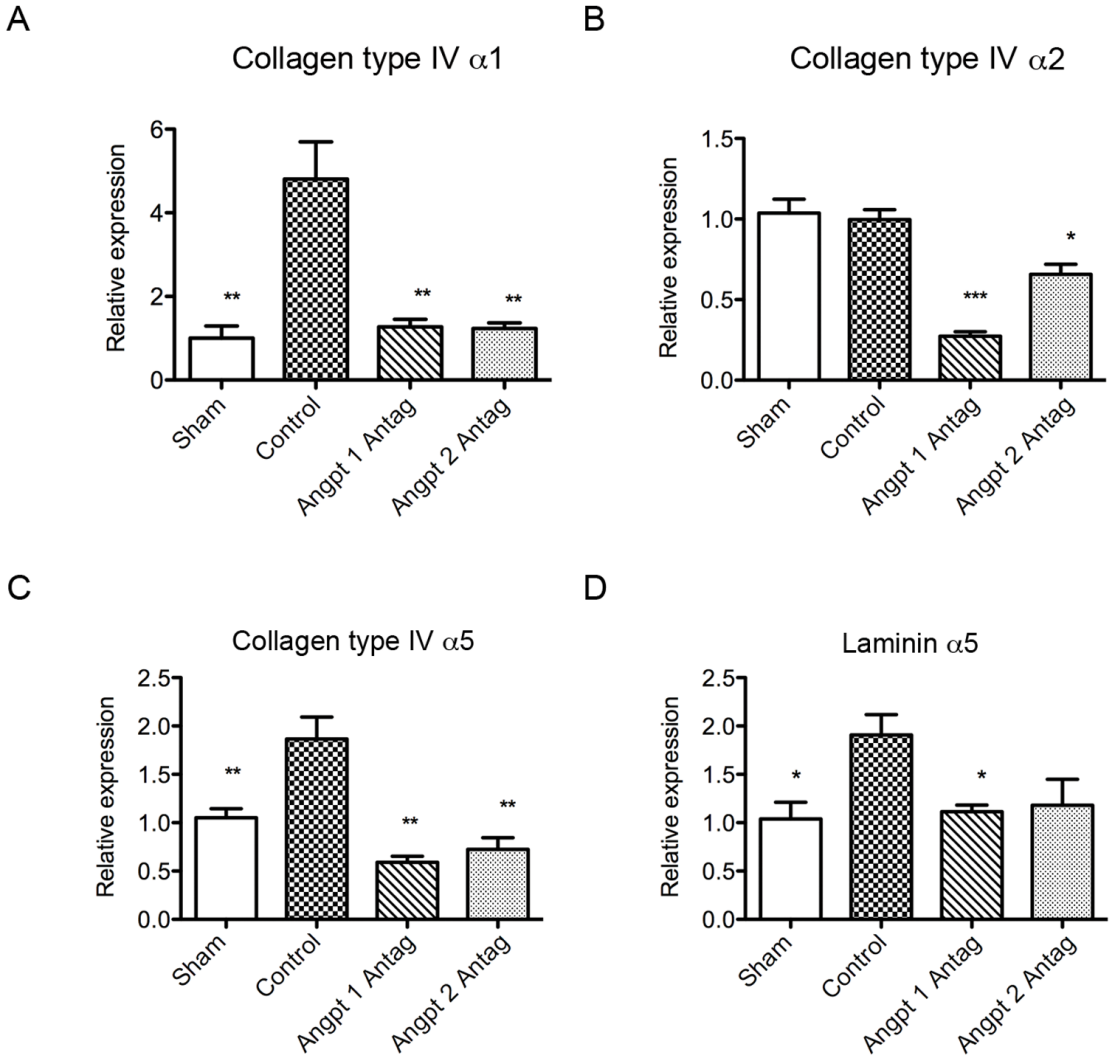
Gene expression analysis of glomerular matrix and basement membrane component proteins with real-time quantitative PCR. (A) Collagen type IV α1 gene expression was higher in the uninephrectomized mice as compared to that in the sham mice. Blocking the action of Angpt 1 or Angpt 2 attenuated the increase in gene expression. (B) No difference in the collagen type IV α2 gene expression was noted between the sham and uninephrectomized group. However, the administration of Angpt 1 or Angpt 2 antagonist decreased its expression to levels lower than those in the sham mice. (C) Uninephrectomy induced up-regulation of glomerular basement membrane collagen type IV α5 gene expression. This effect was attenuated by the Angpt 1 or Angpt 2 blockade. (D) Uninephrectomy also induced the up-regulation of glomerular basement membrane gene laminin α5 expression. The Angpt 1 blockade inhibited the increase in laminin α5 gene expression (* *P*<0.05, **P<0.01, ***P<0.001).

Glomerular gene synthesis in the glomerular basement membrane component was also evaluated using the real-time quantitative PCR method. Uninephrectomy stimulated the genes expression of the major components, including collagen type IV α5 and laminin α5; moreover, the minor glomerular basement membrane component collagen type IV α1 was also upregulated. The Angpt 1 blockade inhibited the increase in the gene expression of collagen type IV α1, α5, and laminin α5, while the Angpt 2 blockade inhibited the increase in the gene expression of glomerular collagen type IV α1 and α5. Although uninephrectomy did not stimulate the gene expression of collagen type IV α2, the Angpt 1 or Angpt 2 blockade downregulated its baseline gene expression to levels lower than even those in the sham mice (Figure 7).

## Discussion

Our present results indicated that Angpt 1 or Angpt 2 may affect glomerular hypertrophy occurring in the late stages following uninephrectomy. Blocking the action of Angpt 1 or Angpt 2, which are important endothelium-related growth factors, probably impaired the neoangiogenesis-like glomerular capillary branching and elongation leading to inhibition of glomerular hypertrophy. This finding was in agreement with those of previous studies, which demonstrated that anti-VEGF administration prevented renal and glomerular hypertrophy. [5], [6], [25] Although blocking Angpt 1 or Angpt 2 influenced the glomerular hypertrophy induced by uninephrectomy, in this study, we did not see any significant proteinuria or glomerular damage associated with impairment in renal function. Electron microscopy also did not reveal any endothelial detachment or glomerular microangiopathy, as observed in the podocyte VEGF knockout mouse model. [26] This finding was somewhat in contrast to the results of Jeansson *et al*. who observed that knocking out Angpt 1 in the embryonic stage of glomerular development resulted in deformed glomeruli with glomerular capillary dilatation and endothelium detachment. [18] It is possible that the absence of Angpt 1 after glomerular maturation is not detrimental to the endothelium in the mature kidney if adequate support of other growth factors—such as VEGF—is available or if detrimental factors are absent. However, Angpt 1 may play a significant role when glomerular capillary remodeling is required or in pathological conditions, such as DM glomerulopathy. [18]

In our study, the administration of antagonists against Angpt 1or Angpt 2 attenuated Tie2 receptor activation in uninephrectomized mice. Angpt 1 is known to activate the Tie2 receptor; therefore, it was clear that Tie2 activation was attenuated by the Angpt 1 antagonist. However, interestingly, Tie2 receptor activation was also attenuated by Angpt 2 antagonist treatment. One explanation is that Angpt 2 may also activate Tie2 receptor during the development of glomerular hypertrophy following uninephrectomy. The traditional view of Angpt 1 and Angpt 2 signaling is that Angpt 2 acts as a naturally occurring antagonist of Angpt 1 on the Tie2 receptor. [9], [10], [27], [28], [29] Recent studies have shown that Angpt 2 can also play an agonistic role, depending on the experimental environment. [30], [31], [32], [33] If expressed at high concentrations or for long durations in cultured endothelial cells, Angpt 2, similar to Angpt 1, can induce Tie2 receptor phosphorylation. [33], [34], [35] Further, Angpt 2 can also promote chemotaxis, tube formation, migration, and sprouting of endothelial cells in the absence of Angpt 1, [33] supporting the view that Angpt 2 actions are context-dependent. The second explanation is that blocking Angpt 2 action reduced the production of glomerular Angpt 1, leading to lower Tie2 receptor activation. This phenomenon may indicate that the manipulation of glomerular endothelium function through an Angpt 2 blockade could influence Angpt 1 production from podocytes during the development of glomerular hypertrophy, since podocytes are currently accepted to be the major source of Angpt 1 in the glomerulus. [11], [12], [13] The glomerular endothelium may secrete certain factors under the influence of Angpt 2, exerting a paracrine function for the secretion of Angpt 1 by podocytes. Alternatively, the development of capillary lengthening or branching itself may stimulate the podocytes to secrete Angpt 1. A disturbance in the capillary remodeling through an Angpt 2 blockade on the endothelium might impair the ability of podocytes to secrete Angpt 1, thereby attenuating the activation of the Tie2 receptor. In our study, the overall glomerular Angpt 1 expression was not significantly different between the control and Angpt 2 blockade group (data not shown). However, the hypothesis cannot be discarded since the whole glomerular Angpt 1 gene (isolated by the sieving method) expression analysis cannot reflect the actual expression in podocyte or its local concentration in the microenvironment where the podocytes and glomerular endothelium are located. Further isolation of podocyte specifically, then analyzing its Angpt 1 gene expression may be helpful to clarify the local expression of the Angpt 1 gene in the microenvironment between podocyte and glomerular endothelium.

Our results also revealed that blocking the action of Angpt 1 or Angpt 2 also influenced the expression of genes related to the glomerular basement membrane, collagen type IV α5, and laminin α5, [36] which was associated with the decrease in the glomerular basement membrane thickness. The glomerular basement membrane collagen type IV α5 is produced by podocytes. [37] Both podocytes and the endothelium express laminin α5 in the mature glomerulus. [38] The downregulation of collagen type IV α5 and laminin α5 is therefore considered to occur be under the influence of the blockade of Angpt 1 or Angpt 2 on the glomerular endothelium and podocytes. Further, although collagen type IV α1 and α2 genes are not the major glomerular basement membrane components in the mature glomerulus, [36], [39] their expression in the glomerulus was also inhibited by the administration of Angpt 1 or Angpt 2 antagonists.

It is interesting that the foot process width was smaller in the Angpt 1 or Angpt 2 antagonist-treated groups. The mechanism underlying this effect is unknown. It appears that interference in glomerular endothelium function through an angiopoietin blockade altered the morphology of podocytes in the uninephrectomy animal model. This phenomenon indicates that the endothelium may exert an influence on podocytes through paracrine functions or that the morphological changes in podocytes are the secondary response to the glomerular capillaries change. In both hypotheses, the influence of the angiopoietin system appears to be involved. Podocytes and glomerular endothelium are located very close to each other, and each may exert its influence on the other in the glomerulus by secreting certain factors via paracrine actions. The systemic effects of the Angpt 1 or Angpt 2 blockade on podocytes may occur via a glomerular endothelium-podocyte interaction. Previous studies have demonstrated that manipulation of the endothelium can influence podocyte behavior, for example, anti-VEGF antibodies and sFlt-1 cause rapid glomerular endothelial cell detachment and hypertrophy, in association with the downregulation of nephrin. [40] Further, Henao *et al.* observed that alterations VEGF expression by podocytes resulted in a dramatic change in the endothelial phenotype and that the subsequent production of endothelin-1 by glomerular endothelium provoked podocyte damage. [41] In our study, manipulating the actions of Angpt 1 or Angpt 2 appeared to influence podocyte hypertrophy and foot process width, and altered the gene expression of collagen and laminin in podocytes. All these changes may occur because of alterations in an endothelium-derived factor, which is currently unknown, following the Angpt 1or Angpt 2 blockade.

However, our study was limited by the fact that the electron microscopic sampling study was not extensive. A bias in the measurement may also have occurred since variations in the width or thickness of foot processes and basement membrane measurements are typically high. A more extensive studies are mandatory.

Our findings demonstrate that the angiopoietin system plays an important role in physiological glomerular hypertrophy after uninephrectomy. In addition to its actions on the endothelium, angiopoietin may also exert an influence on other glomerular component cells through endothelial paracrine functions or secondary to the endothelium-capillary change. Taken together with the findings of previous studies by other authors, these results indicate that an imbalance in the angiopoietin system may play a detrimental role in the development of glomerular damage, suggesting that the correction of such an imbalance may be helpful in treating glomerular diseases. To test these hypotheses, it is mandatory to use conditionally Angpt 1 or 2 knock out (or over expression transgenic) mice or agonist treatment experiments to clarify their roles in glomerular pathophysiology.

## References

[1] Nyengaard JR (1993) Number and dimensions of rat glomerular capillaries in normal development and after nephrectomy. Kidney Int 43: 1049–1057. 8510382 10.1038/ki.1993.147

[2] Nyengaard JR, Rasch R (1993) The impact of experimental diabetes mellitus in rats on glomerular capillary number and sizes. Diabetologia 36: 189–194. 8462766 10.1007/BF00399948

[3] Marcussen N, Nyengaard JR, Christensen S (1994) Compensatory growth of glomeruli is accomplished by an increased number of glomerular capillaries. Laboratory investigation; a journal of technical methods and pathology 70: 868–874. 8015291

[4] Flyvbjerg A, Schrijvers BF, De Vriese AS, Tilton RG, Rasch R (2002) Compensatory glomerular growth after unilateral nephrectomy is VEGF dependent. American journal of physiology Endocrinology and metabolism 283: E362–366. 12110543 10.1152/ajpendo.00007.2002

[5] Schrijvers BF, Flyvbjerg A, Tilton RG, Lameire NH, De Vriese AS (2006) A neutralizing VEGF antibody prevents glomerular hypertrophy in a model of obese type 2 diabetes, the Zucker diabetic fatty rat. Nephrology, dialysis, transplantation : official publication of the European Dialysis and Transplant Association - European Renal Association 21: 324–329. 10.1093/ndt/gfi217 16249198

[6] Schrijvers BF, Rasch R, Tilton RG, Flyvbjerg A (2002) High protein-induced glomerular hypertrophy is vascular endothelial growth factor-dependent. Kidney international 61: 1600–1604. 11967009 10.1046/j.1523-1755.2002.00310.x

[7] Morisada T, Kubota Y, Urano T, Suda T, Oike Y (2006) Angiopoietins and angiopoietin-like proteins in angiogenesis. Endothelium : journal of endothelial cell research 13: 71–79. 16728326 10.1080/10623320600697989

[8] Lobov IB, Brooks PC, Lang RA (2002) Angiopoietin-2 displays VEGF-dependent modulation of capillary structure and endothelial cell survival in vivo. Proceedings of the National Academy of Sciences of the United States of America 99: 11205–11210. 12163646 10.1073/pnas.172161899 PMC123234

[9] Maisonpierre PC, Suri C, Jones PF, Bartunkova S, Wiegand SJ, et al (1997) Angiopoietin-2, a natural antagonist for Tie2 that disrupts in vivo angiogenesis. Science 277: 55–60. 9204896 10.1126/science.277.5322.55

[10] Suri C, Jones PF, Patan S, Bartunkova S, Maisonpierre PC, et al (1996) Requisite role of angiopoietin-1, a ligand for the TIE2 receptor, during embryonic angiogenesis. Cell 87: 1171–1180. 8980224 10.1016/s0092-8674(00)81813-9

[11] Campean V, Karpe B, Haas C, Atalla A, Peters H, et al (2008) Angiopoietin 1 and 2 gene and protein expression is differentially regulated in acute anti-Thy1.1 glomerulonephritis. Am J Physiol Renal Physiol 294: F1174–1184. 18272601 10.1152/ajprenal.00320.2007

[12] Woolf AS, Yuan HT (2001) Angiopoietin growth factors and Tie receptor tyrosine kinases in renal vascular development. Pediatr Nephrol 16: 177–184. 11261688 10.1007/s004670000509

[13] Yuan HT, Suri C, Yancopoulos GD, Woolf AS (1999) Expression of angiopoietin-1, angiopoietin-2, and the Tie-2 receptor tyrosine kinase during mouse kidney maturation. J Am Soc Nephrol 10: 1722–1736. 10446940 10.1681/ASN.V1081722

[14] Holash J, Maisonpierre PC, Compton D, Boland P, Alexander CR, et al (1999) Vessel cooption, regression, and growth in tumors mediated by angiopoietins and VEGF. Science 284: 1994–1998. 10373119 10.1126/science.284.5422.1994

[15] Dumont DJ, Gradwohl G, Fong GH, Puri MC, Gertsenstein M, et al (1994) Dominant-negative and targeted null mutations in the endothelial receptor tyrosine kinase, tek, reveal a critical role in vasculogenesis of the embryo. Genes Dev 8: 1897–1909. 7958865 10.1101/gad.8.16.1897

[16] Thurston G, Rudge JS, Ioffe E, Zhou H, Ross L, et al (2000) Angiopoietin-1 protects the adult vasculature against plasma leakage. Nat Med 6: 460–463. 10742156 10.1038/74725

[17] Yuan HT, Suri C, Yancopoulos GD, Woolf AS (1999) Expression of angiopoietin-1, angiopoietin-2, and the Tie-2 receptor tyrosine kinase during mouse kidney maturation. Journal of the American Society of Nephrology : JASN 10: 1722–1736. 10446940 10.1681/ASN.V1081722

[18] Jeansson M, Gawlik A, Anderson G, Li C, Kerjaschki D, et al (2011) Angiopoietin-1 is essential in mouse vasculature during development and in response to injury. The Journal of clinical investigation 121: 2278–2289. 21606590 10.1172/JCI46322 PMC3104773

[19] Falcon BL, Hashizume H, Koumoutsakos P, Chou J, Bready JV, et al (2009) Contrasting actions of selective inhibitors of angiopoietin-1 and angiopoietin-2 on the normalization of tumor blood vessels. The American journal of pathology 175: 2159–2170. 19815705 10.2353/ajpath.2009.090391 PMC2774078

[20] Reinhardt C, Bergentall M, Greiner TU, Schaffner F, Ostergren-Lunden G, et al (2012) Tissue factor and PAR1 promote microbiota-induced intestinal vascular remodelling. Nature 483: 627–631. 22407318 10.1038/nature10893 PMC3885420

[21] Coxon A, Bready J, Min H, Kaufman S, Leal J, et al (2010) Context-dependent role of angiopoietin-1 inhibition in the suppression of angiogenesis and tumor growth: implications for AMG 386, an angiopoietin-1/2-neutralizing peptibody. Mol Cancer Ther 9: 2641–2651. 20937592 10.1158/1535-7163.MCT-10-0213 PMC4430860

[22] Tressel SL, Kim H, Ni CW, Chang K, Velasquez-Castano JC, et al (2008) Angiopoietin-2 stimulates blood flow recovery after femoral artery occlusion by inducing inflammation and arteriogenesis. Arterioscler Thromb Vasc Biol 28: 1989–1995. 18772493 10.1161/ATVBAHA.108.175463 PMC2613177

[23] Mundel P, Reiser J, Zuniga Mejia Borja A, Pavenstadt H, Davidson GR, et al (1997) Rearrangements of the cytoskeleton and cell contacts induce process formation during differentiation of conditionally immortalized mouse podocyte cell lines. Experimental cell research 236: 248–258. 9344605 10.1006/excr.1997.3739

[24] Weibel ER (1979) Stereological Methods: Practical Methods for Biological Morphometiy. London: Academic Press. 51–57 p.

[25] Schrijvers BF, Flyvbjerg A, Tilton RG, Rasch R, Lameire NH, et al (2005) Pathophysiological role of vascular endothelial growth factor in the remnant kidney. Nephron Experimental nephrology 101: e9–15. 15925906 10.1159/000086034

[26] Eremina V, Jefferson JA, Kowalewska J, Hochster H, Haas M, et al (2008) VEGF inhibition and renal thrombotic microangiopathy. The New England journal of medicine 358: 1129–1136. 18337603 10.1056/NEJMoa0707330 PMC3030578

[27] Davis S, Aldrich TH, Jones PF, Acheson A, Compton DL, et al (1996) Isolation of angiopoietin-1, a ligand for the TIE2 receptor, by secretion-trap expression cloning. Cell 87: 1161–1169. 8980223 10.1016/s0092-8674(00)81812-7

[28] Holash J, Wiegand SJ, Yancopoulos GD (1999) New model of tumor angiogenesis: dynamic balance between vessel regression and growth mediated by angiopoietins and VEGF. Oncogene 18: 5356–5362. 10498889 10.1038/sj.onc.1203035

[29] Kim KE, Cho CH, Kim HZ, Baluk P, McDonald DM, et al (2007) In vivo actions of angiopoietins on quiescent and remodeling blood and lymphatic vessels in mouse airways and skin. Arterioscler Thromb Vasc Biol 27: 564–570. 17194894 10.1161/01.ATV.0000256458.82320.be

[30] Gale NW, Thurston G, Hackett SF, Renard R, Wang Q, et al (2002) Angiopoietin-2 is required for postnatal angiogenesis and lymphatic patterning, and only the latter role is rescued by Angiopoietin-1. Dev Cell 3: 411–423. 12361603 10.1016/s1534-5807(02)00217-4

[31] Kim I, Kim JH, Moon SO, Kwak HJ, Kim NG, et al (2000) Angiopoietin-2 at high concentration can enhance endothelial cell survival through the phosphatidylinositol 3'-kinase/Akt signal transduction pathway. Oncogene 19: 4549–4552. 11002428 10.1038/sj.onc.1203800

[32] Bogdanovic E, Nguyen VP, Dumont DJ (2006) Activation of Tie2 by angiopoietin-1 and angiopoietin-2 results in their release and receptor internalization. J Cell Sci 119: 3551–3560. 16895971 10.1242/jcs.03077

[33] Korff T, Kimmina S, Martiny-Baron G, Augustin HG (2001) Blood vessel maturation in a 3-dimensional spheroidal coculture model: direct contact with smooth muscle cells regulates endothelial cell quiescence and abrogates VEGF responsiveness. FASEB J 15: 447–457. 11156960 10.1096/fj.00-0139com

[34] Teichert-Kuliszewska K, Maisonpierre PC, Jones N, Campbell AI, Master Z, et al (2001) Biological action of angiopoietin-2 in a fibrin matrix model of angiogenesis is associated with activation of Tie2. Cardiovasc Res 49: 659–670. 11166279 10.1016/s0008-6363(00)00231-5

[35] Yuan HT, Khankin EV, Karumanchi SA, Parikh SM (2009) Angiopoietin 2 is a partial agonist/antagonist of Tie2 signaling in the endothelium. Mol Cell Biol 29: 2011–2022. 19223473 10.1128/MCB.01472-08 PMC2663314

[36] Miner JH (1998) Developmental biology of glomerular basement membrane components. Curr Opin Nephrol Hypertens 7: 13–19. 9442357 10.1097/00041552-199801000-00003

[37] Abrahamson DR, Hudson BG, Stroganova L, Borza DB, St John PL (2009) Cellular origins of type IV collagen networks in developing glomeruli. J Am Soc Nephrol 20: 1471–1479. 19423686 10.1681/ASN.2008101086 PMC2709682

[38] St John PL, Abrahamson DR (2001) Glomerular endothelial cells and podocytes jointly synthesize laminin-1 and -11 chains. Kidney Int 60: 1037–1046. 11532098 10.1046/j.1523-1755.2001.0600031037.x

[39] Miner JH (1999) Renal basement membrane components. Kidney international 56: 2016–2024. 10594777 10.1046/j.1523-1755.1999.00785.x

[40] Sugimoto H, Hamano Y, Charytan D, Cosgrove D, Kieran M, et al (2003) Neutralization of circulating vascular endothelial growth factor (VEGF) by anti-VEGF antibodies and soluble VEGF receptor 1 (sFlt-1) induces proteinuria. The Journal of biological chemistry 278: 12605–12608. 12538598 10.1074/jbc.C300012200

[41] Henao DE, Saleem MA, Cadavid AP (2010) Glomerular disturbances in preeclampsia: disruption between glomerular endothelium and podocyte symbiosis. Hypertens Pregnancy 29: 10–20. 19263286 10.3109/10641950802631036

